# Beyond Recovery: Effects of Post-Exercise Milk and Milk-Based Beverages on Appetite Regulation and Energy Intake—A Systematic Review and Meta-Analysis

**DOI:** 10.3390/nu18111656

**Published:** 2026-05-22

**Authors:** Elif Tunçil, Yiğitcan Karanfil, Emre Dünder

**Affiliations:** 1 Department of Nutrition and Dietetics, Faculty of Health Sciences, Hacettepe University, 06100 Ankara, Türkiye; 2 Department of Nutrition and Dietetics, Faculty of Health Sciences, Balıkesir University, 10145 Balıkesir, Türkiye; 3 Department of Sports Medicine, Faculty of Medicine, Hacettepe University, 06100 Ankara, Türkiye; yigitcan.karanfil@hacettepe.edu.tr; 4 Department of Statistics, Faculty of Sciences, Ondokuz Mayıs University, 55200 Samsun, Türkiye; emre.dunder@omu.edu.tr

**Keywords:** milk, milk-based beverage, appetite, energy intake, post-exercise

## Abstract

Background/Objectives: Milk and milk-based beverages have shown potential benefits for maintaining exercise-induced negative energy balance. However, this has not been systematically investigated. Therefore, this review aimed to evaluate the effects of post-exercise milk or milk-based beverages consumption on appetite regulation and energy intake. Methods: A comprehensive search was conducted in PubMed, Scopus, Web of Science, the Cochrane Library, Ovid MEDLINE ALL, Open Access Theses and Dissertations, and EBSCO Open Dissertations up to 6 April 2025. Eligible studies were randomized controlled trials assessing the effects of milk or milk-based beverages on post-exercise appetite regulation in healthy adults. Study selection, data extraction, and risk of bias assessment (RoB-2) were performed independently by two reviewers. Meta-analysis was conducted where appropriate using mean differences with 95% confidence intervals (CI). Subgroup analyses were conducted by sex and intervention. Results: Twelve studies (*n* = 140) were included, of which 10 (*n* = 118) contributed to the meta-analysis of energy intake. Milk and milk-based beverages were associated with lower energy intake than carbohydrate (CHO) beverages (−72.73 kcal, 95% CI [−141.69; −3.77]; *I*^2^ = 0%, *p* = 0.039). Subgroup analyses indicated no effect modification by sex or intervention type. For subjective appetite ratings (11 studies, *n* = 125), meta-analysis was not performed due to measurement and reporting heterogeneity, and no clear differences or only mild appetite-suppressive effects were observed. Appetite-related hormones were assessed in two studies (*n* = 23), with no overlapping outcomes. Conclusions: Post-exercise consumption of milk and milk-based beverages may reduce energy intake compared with CHO beverages, although effects on subjective appetite are inconsistent and evidence for hormonal responses remains limited.

## 1. Introduction

The post-exercise period represents a critical window for optimizing recovery, facilitating physiological adaptation, and supporting subsequent performance [1]. Nutritional strategies applied during this phase are designed to stimulate muscle protein synthesis, resynthesize muscle glycogen, and restore fluid and electrolyte balance following exercise-induced losses [2,3]. These processes are essential for maintaining training quality, especially when recovery time between exercise sessions is restricted. Current sports nutrition guidelines recommend that nutrient intake should occur soon after exercise, with the first 2 to 4 h considered a key window for effective recovery [4,5,6]. Within this period, adequate consumption of carbohydrate (CHO), protein, fluids, and electrolytes is emphasized to support these recovery processes [2,4].

Milk has been proposed as an effective recovery beverage due to its naturally occurring combination of nutrients that are consistent with the nutritional demands of recovery [7,8,9]. It supplies fluids and electrolytes, along with CHO and high-quality proteins such as whey and casein [10]. This composition reflects current post-exercise recommendations, with CHO intake supporting glycogen resynthesis and protein intake promoting muscle protein synthesis, while combined intake may be beneficial when CHO intake is insufficient [4]. In addition, the electrolyte content of milk, including sodium and potassium, may enhance fluid retention and contribute to effective rehydration by supporting electrolyte balance, reducing urine output, and promoting the restoration of fluid balance [11,12].

Several studies have explored how milk and milk-based beverages influence post-exercise recovery [13,14,15,16,17,18,19,20,21,22,23]. Evidence suggests that milk and milk-based beverages may provide similar, or in some cases potentially superior, recovery benefits compared with alternative recovery beverages. Experimental studies have mainly focused on their role in muscle protein synthesis [13], exercise-induced muscle damage [14,15,16], glycogen resynthesis [17,18], rehydration [19,20], inflammatory responses [21], and subsequent exercise performance [22,23]. Beyond these recovery outcomes, milk and milk-based beverages have also been investigated for their potential influence on appetite regulation and subsequent energy intake [24,25,26]. These effects are particularly important for individuals aiming to reduce body weight or body fat, as appetite regulation may influence the ability to sustain an exercise-induced negative energy balance. In this regard, some studies have reported reduced hunger, increased fullness, and/or lower subsequent energy intake following milk or milk-based beverages compared with isocaloric or non-caloric control beverages, whereas others have observed no significant differences across these outcomes [24,25,26]. These findings indicate that how milk and milk-based beverages influence appetite regulation and energy intake may be context-dependent and not yet fully understood. The variability in study designs, outcome criteria, and reporting approaches underscores the need for a more comprehensive and systematic synthesis of the existing literature.

Although interest in the use of milk for post-exercise recovery has grown, relatively few systematic reviews and meta-analyses have specifically evaluated milk or milk-based beverages within this context [8,27,28,29,30,31]. Existing systematic reviews and meta-analyses have mainly examined muscle protein synthesis [27], glycogen resynthesis [27,28], hydration [27,29], recovery biomarkers [8,30,31], and subsequent exercise performance [8,27,28,30]. However, to date, the role of milk and milk-based beverages in post-exercise appetite regulation and subsequent energy intake has not been systematically synthesized. Therefore, this systematic review and meta-analysis aimed to examine post-exercise milk and milk-based beverage consumption with respect to subjective appetite ratings, appetite-related hormones, and subsequent energy intake in healthy adults.

## 2. Materials and Methods

This systematic review was conducted in accordance with the Preferred Reporting Items for Systematic Reviews and Meta-Analyses (PRISMA 2020) guidelines [32] and registered in the International Prospective Register of Systematic Reviews (PROSPERO) (CRD420251032252).

### 2.1. Eligibility Criteria

Eligibility criteria were established using the population, intervention, comparator, outcomes, and study design (PICOS) framework. The detailed inclusion and exclusion criteria according to each PICOS component are summarized in Table 1. Briefly, studies were eligible if they included healthy adult participants, investigated the effects of post-exercise consumption of milk or milk-based beverages, and reported outcomes related to subjective appetite, appetite-related hormones, or energy intake. Only randomized study designs were included. Studies involving participants with diagnosed clinical conditions, non-randomized study designs, animal studies, and in vitro experiments were excluded. In addition, only studies investigating post-exercise beverage consumption were included, whereas studies examining intake before or during exercise were excluded.

### 2.2. Search Strategy

A comprehensive literature search was conducted in PubMed, Scopus, Web of Science, the Cochrane Library (CENTRAL), and Ovid MEDLINE ALL. Gray literature sources were also searched, including Open Access Theses and Dissertations and EBSCO Open Dissertations. The reference lists of relevant articles were also screened to identify additional eligible studies. Medical Subject Headings (MeSH) terms and free-text terms were used in the search strategy (Supplementary Materials S1). The search strategy included the following terms: (Milk OR “Dairy Products” OR “Milk protein” OR Whey OR Protein*) AND (Exercise OR “Post exercise” OR “Post-exercise” OR Exertion OR Fitness OR Activ* OR Sport* OR Train* OR “Post-Exercise Recovery” OR Workout)) AND (Appetite OR “Appetite Regulation” OR Satiety OR Hunger OR Fullness OR “Energy Intake” OR “Energy Balance”)) AND (intervention OR “intervention* stud*” OR “controlled trial*” OR random* OR placebo OR clinical OR “clinical trial*” OR trial* OR “randomi?ed controlled trial*” OR “randomi?ed clinical trial*” OR RCT OR blinded OR “double-blind*” OR “double blind*” OR “Cross over” OR “Cross-Over” OR “parallel study” OR “parallel trial”). No language or date restrictions were applied. Detailed search strategies for all databases, including the search dates and the number of records identified, are provided in Supplementary Materials S1.

### 2.3. Study Selection

All records were imported into Rayyan systematic review software (version 1.7.5) for study management and screening [33]. Potential duplicate records were identified by the software and subsequently verified through manual review by two independent reviewers. No records were automatically excluded. After duplicate removal, titles and abstracts were screened independently by two reviewers in blind mode using Rayyan against the predefined eligibility criteria. Potentially relevant studies were then assessed through full text review. Disagreements between reviewers were resolved through discussion, and when consensus could not be reached, a third reviewer was consulted.

### 2.4. Data Extraction

Data from the included studies were extracted independently by two reviewers using a predefined data extraction form developed in Microsoft Excel (Microsoft Office Professional Plus 2016). Discrepancies between reviewers were resolved through discussion, and when necessary, consultation with a third reviewer. The following information was extracted from each study: author and year of publication, study design, participant characteristics, exercise type, characteristics of the milk or milk-based beverage intervention, characteristics of the control beverage(s), and reported outcomes. Outcomes of interest included energy intake, subjective appetite ratings, and appetite-related hormones. For studies reporting multiple post-exercise time points, all relevant time points were extracted and considered during data synthesis. Numerical data required for quantitative synthesis (e.g., means, standard deviations, and sample sizes) were extracted whenever available. In cases where outcomes were reported using different units, scales, or heterogeneous metrics (e.g., area under the curve values or only *p*-values), data were extracted as reported and synthesized narratively if standardization was not feasible.

### 2.5. Risk of Bias and Certainty of Evidence

Risk of bias was assessed using the Cochrane Risk of Bias 2 (RoB 2) tool for randomized trials (crossover trials version, Beta V2, revised 18 March 2021; Excel macro-enabled assessment tool) [34]. Following the Cochrane Handbook for Systematic Reviews of Interventions and the guidance for the RoB 2 tool, risk of bias was assessed at the level of individual outcomes rather than at the study level [34,35]. Assessments were conducted by two independent reviewers, and disagreements were resolved through discussion or consultation with a third reviewer. Risk of bias was categorized as low risk, some concerns, or high risk.

The certainty of evidence for each outcome was evaluated using the Grading of Recommendations Assessment, Development and Evaluation (GRADE) approach [36]. Evidence was rated across the domains of risk of bias, inconsistency, indirectness, imprecision, and publication bias, and classified as high, moderate, low, or very low certainty.

### 2.6. Data Synthesis and Statistical Analysis

Findings were synthesized narratively and organized by outcome domain. When at least two studies reported comparable outcomes with sufficient numerical data, a meta-analysis was performed. For continuous outcomes, pooled effect sizes were calculated as mean differences (MD) or standardized mean differences (SMD), depending on the measurement scale, with corresponding 95% confidence intervals (CI). Energy intake values reported in different units were converted to kilocalories (kcal) prior to analysis. Whenever necessary, standard deviations (SD) were calculated from standard errors of the mean (SEM) or confidence intervals using standard formulas [35].

Statistical heterogeneity was assessed using Cochran’s Q test and quantified using the *I*^2^ statistic, with values of 25%, 50%, and 75% representing low, moderate, and high heterogeneity, respectively [37]. The meta-analytic model was selected according to the level of heterogeneity. A fixed-effect model was used when heterogeneity was negligible, while a random-effects model was applied when substantial heterogeneity was present. Meta-analyses were conducted using the inverse variance method, and results were presented using forest plots.

In anticipation of potential heterogeneity, predefined subgroup analyses were planned based on sex and intervention type (milk vs. milk-based beverages). Differences between subgroups were assessed using the chi-square test. Sensitivity analyses were conducted to assess the robustness of the overall findings by excluding studies at high risk of bias or with methodological inconsistencies (i.e., non-standardized intervention characteristics) and recalculating pooled effect sizes using the same meta-analytic model. Publication bias was assessed through visual inspection of funnel plots and formally evaluated using Egger’s regression test [38] and Begg and Mazumdar’s rank correlation test [39]. A two-sided *p*-value < 0.05 was considered statistically significant. All statistical analyses were conducted using R statistical software (R Core Team, version 4.5.0, 2025 Vienna, Austria).

## 3. Results

### 3.1. Study Selection

**Figure 1:**
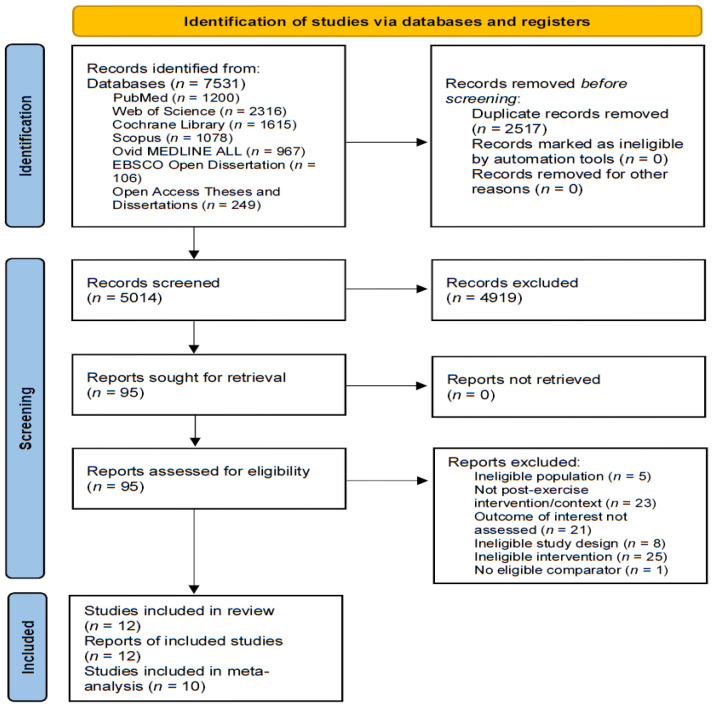
PRISMA flow diagram.

Through database searching, 7531 records were identified. After removing 2517 duplicates, 5014 records were screened by title and abstract. Of these, 4919 were excluded, leaving 95 reports for full-text assessment. All full-text reports were obtained and assessed for eligibility. After full-text evaluation, 83 reports were excluded for the following reasons: ineligible intervention (*n* = 25), not post-exercise intervention/context (*n* = 23), outcome of interest not assessed (*n* = 21), ineligible study design (*n* = 8), ineligible population (*n* = 5), and no eligible comparator (*n* = 1). The systematic review included 12 studies [24,25,26,40,41,42,43,44,45,46,47,48], and data from 10 of these were sufficient for inclusion in the meta-analysis [24,25,26,41,43,44,45,46,47,48]. Figure 1 presents the study selection process according to the PRISMA flow diagram.

### 3.2. Study Characteristics

The main characteristics of the included studies are summarized in Table 2. All studies were randomized crossover trials with acute interventions, published between 2014 and 2023. A total of 140 participants were included, comprising 90 males (8 studies) [25,40,41,42,43,44,45,48] and 50 females (4 studies) [24,26,46,47]. Participants were mainly young adults (mean age across 11 studies: 24 ± 4 years), whereas one study [44] included older participants (64 ± 3 years). Physical activity levels were predominantly recreational. Exercise protocols were predominantly endurance-based, with cycling as the primary modality (9 studies), while the remainder used resistance or aerobic exercise.

Interventions consisted of milk, milk-based beverages, whey-based drinks, or high-protein milk, with milk being the most commonly used intervention. The protein content ranged from 13 to 115 g, and beverage volumes varied widely (250 mL to 2.7 L). Comparator conditions primarily consisted of energy-containing CHO beverages, with several studies also including a non-caloric placebo (typically water). In most studies, intervention and comparator beverages were isovolumetric and isocaloric [24,26,41,45,47,48]. In two studies, beverage volumes were determined based on body mass loss during exercise [40,42], and in two others, beverages were provided ad libitum alongside meals, resulting in non-standardized beverage volumes [25,46]. Wan et al. (2018) used isovolumetric but not isocaloric beverages [43], whereas Maltais et al. (2018) used beverages that were closely matched in both volume and energy content, although not identical [44].

### 3.3. Risk of Bias

**Figure 2:**
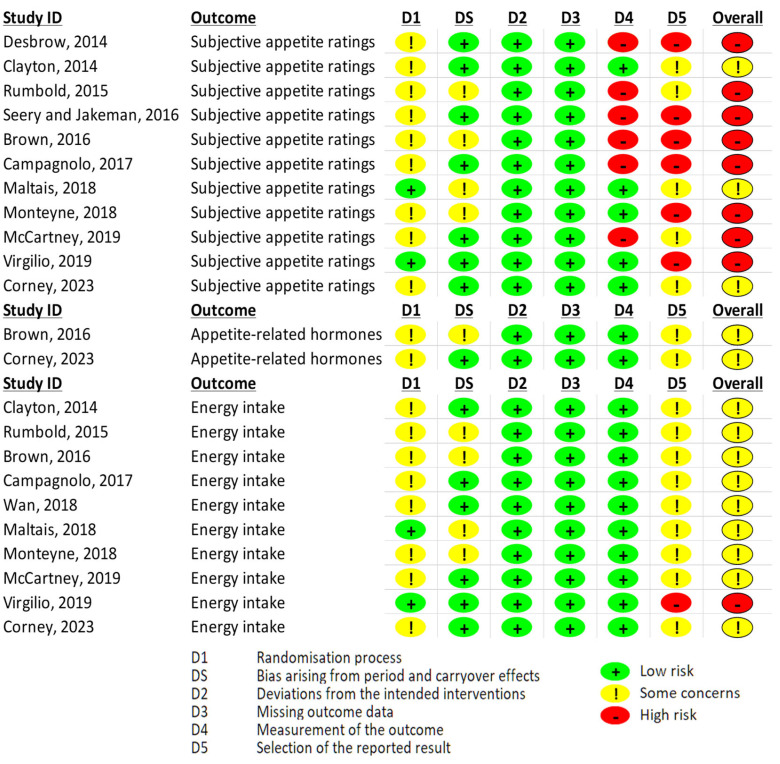
Outcome-based traffic light plot of risk of bias [24,25,26,40,41,42,43,44,45,46,47,48].

Figure 2 displays the findings of the risk of bias assessment by outcome. Overall, most studies were assessed as having some concerns; the main reasons included insufficient reporting of allocation concealment and the lack of clearly defined pre-specified analysis plans. Given the crossover design of all included studies, carryover effects were considered through evaluation of washout periods. Washout duration was deemed adequate in all but one study, for which insufficient information was reported [24]. Blinding procedures varied across studies and contributed to risk of bias, particularly in relation to outcome measurement. Four studies were reported as double-blind [41,44,45,47]. In one study, blinding was not explicitly stated; however, beverages were prepared with similar flavor profiles, suggesting an attempt to minimize detection bias [48]. In the remaining studies, beverages differed in taste and sensory characteristics, making participant blinding unlikely and increasing the potential for bias, especially in subjective outcomes [24,25,26,40,42,43,46]. Beyond the absence of blinding, the high risk of bias in subjective appetite ratings was largely attributable to the incomplete reporting of multiple measurements. Studies evaluating energy intake were predominantly judged as having some concerns, with only one classified as high risk of bias as a result of post hoc subgroup analyses that were not clearly pre-specified [47]. Evidence for appetite-related hormones was limited, and all contributing studies were judged as having some concerns.

### 3.4. Effects on Subjective Appetite

Subjective appetite ratings were assessed in 11 studies (*n* = 125) [24,25,26,40,41,42,44,45,46,47,48] via visual analog scales (VAS), capturing at least one of the following dimensions: hunger, fullness, desire to eat, and prospective food consumption (PFC), typically measured at multiple time points. Across studies, hunger was evaluated in all 11 studies, with 4 reporting significantly lower scores following milk or milk-based beverages compared with control conditions, while the remaining 7 reported no significant differences. Fullness was assessed in 10 studies, of which 3 reported significantly higher scores following milk or milk-based beverages, whereas 7 found no significant effects. PFC was examined in 6 studies, with only one study reporting significantly lower values following milk-based beverages compared with placebo. Desire to eat was assessed in 4 studies, none of which reported significant differences between conditions.

A meta-analysis could not be performed because of substantial heterogeneity in outcome reporting, including differences in measurement scales (e.g., 100 mm vs. 150 mm VAS), timing of assessments, and statistical reporting formats (e.g., mm values vs. area under the curve), as well as incomplete reporting of numerical data, with most studies reporting only *p*-values. Overall, post-exercise milk or milk-based beverages did not produce consistent changes in subjective appetite outcomes compared with control conditions; however, when effects were observed, they were generally in the direction of appetite suppression.

### 3.5. Effects on Appetite-Related Hormones

Appetite-related hormones were assessed in 2 studies (*n* = 23), examining leptin, insulin, acylated ghrelin, and glucagon-like peptide-1 (GLP-1) [24,48]. In one study, milk-based beverages resulted in significantly higher post-exercise insulin and GLP-1 concentrations compared with placebo, while leptin showed no significant differences [24]. In the other study, acylated ghrelin levels did not differ significantly between conditions [48]. A meta-analysis was not performed, as the number of available studies was limited and no common outcomes were reported. Each hormone was assessed in only a single study, precluding quantitative synthesis, as at least two independent data points are required for meta-analysis. Furthermore, heterogeneity in the hormonal outcomes limited comparability across studies.

### 3.6. Effects on Energy Intake

Energy intake was assessed in 10 studies (*n* = 118) [24,25,26,41,43,44,45,46,47,48]. Energy intake was most commonly determined using ad libitum meal protocols, whereas two studies assessed intake under fully free-living conditions using dietary records [43,47]. Beverage volumes were standardized across studies, except for two trials in which beverages were also consumed ad libitum [25,46]. As energy intake outcomes could be influenced by the energy content of beverages, comparisons were restricted to active control conditions (CHO beverages) rather than non-caloric placebos. To account for differences in the energy content of beverages, outcomes were evaluated as total energy intake, including both beverage and subsequent food intake. Across studies, post-exercise consumption of milk or milk-based beverages resulted in lower energy intake in 3 studies, while 7 studies reported no statistically significant differences compared with CHO beverages. Despite the lack of statistical significance in most studies, energy intake tended to be lower following milk or milk-based beverages, ranging from 2% to 25% (−9 to −304 kcal) compared with CHO beverages.

**Figure 3:**
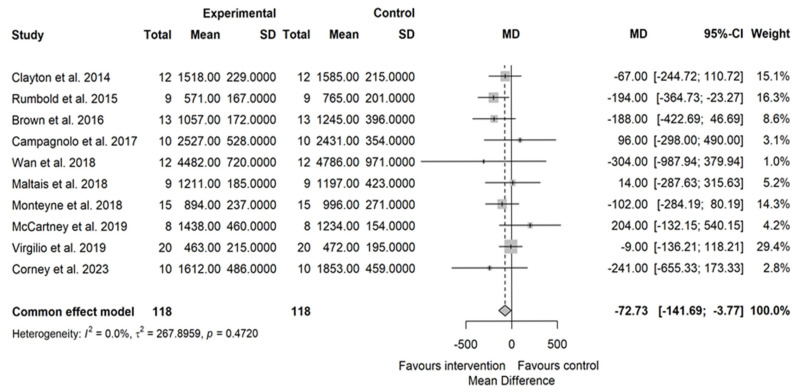
Forest plot of the effect of milk and milk-based beverages on energy intake [24,25,26,41,43,44,45,46,47,48].

Heterogeneity across studies was assessed prior to meta-analysis. Cochran’s Q test was not statistically significant (Q = 8.63, *df* = 9, *p* = 0.47), and the *I*^2^ statistic was 0.0% (95% CI [0%; 62.4%]), indicating no evidence of heterogeneity. Based on these findings, a fixed-effect model was applied in the meta-analysis. There was a small but statistically significant reduction in energy intake following the consumption of milk or milk-based beverages compared with CHO beverages (MD: −72.73 kcal [95% CI: −141.69 to −3.77], z = −2.07, *p* = 0.039). Figure 3 illustrates the forest plot of the pooled analysis.

#### 3.6.1. Subgroup Analysis

Subgroup analyses were conducted to examine whether the effects of milk and milk-based beverages on post-exercise energy intake differed according to sex (male vs. female) and intervention type (milk vs. milk-based beverage). No statistically significant effects were observed within either sex subgroup (Figure S1). The pooled mean difference was −74.28 kcal (95% CI: −181.37 to 32.82; *I*^2^ = 0%) in males (6 studies) and −71.64 kcal (95% CI: −161.77 to 18.49; *I*^2^ = 53.4%) in females (4 studies). The test for subgroup differences was not statistically significant (χ^2^ = 0.00, *df* = 1, *p* = 0.971), indicating no evidence of a differential effect by sex. Similarly, no statistically significant effects were observed according to intervention type (Figure S2). The pooled mean difference was −57.67 kcal (95% CI: −139.82 to 24.48; *I*^2^ = 0%) for milk-based beverages (5 studies) and −108.68 kcal (95% CI: −235.56 to 18.21; *I*^2^ = 28.9%) for milk (5 studies). The between-subgroup difference was not statistically significant (χ^2^ = 0.44, *df* = 1, *p* = 0.508), suggesting no clear difference between intervention types.

#### 3.6.2. Sensitivity Analysis

Among the included studies, one study was judged to be at high risk of bias [47], and two studies used ad libitum beverage protocols rather than standardized serving volumes [25,46]. Sensitivity analyses were therefore conducted to assess the robustness of the primary meta-analysis of energy intake. Exclusion of the high risk of bias study resulted in a larger pooled effect size, which remained statistically significant (MD = −99.26 kcal, 95% CI: −181.32 to −17.19; z = −2.37, *p* = 0.017). Exclusion of the two studies employing ad libitum beverage protocols also yielded a statistically significant effect, with a slightly greater magnitude (MD = −90.87 kcal, 95% CI: −165.77 to −18.55; z = −2.41, *p* = 0.016). Across all analyses, including the primary model, the direction of effect remained consistent, with a slightly greater magnitude observed in both sensitivity analyses.

### 3.7. Publication Bias

Visual inspection of the funnel plot indicated an approximately symmetrical distribution of effect sizes around the pooled estimate, with no clear indication of asymmetry (Figure S3). Studies were distributed on both sides of the overall effect size, with no apparent pattern suggesting that smaller studies reported systematically different effects. Egger’s regression test did not indicate funnel plot asymmetry (t = −0.05, *df* = 8, *p* = 0.962), and similar results were observed with Begg and Mazumdar’s rank correlation test (z = 0.09, *p* = 0.929). Overall, these findings did not indicate publication bias in the analysis of energy intake.

### 3.8. Certainty of Evidence

The summary of findings and GRADE assessments are presented in Table 3. Overall, certainty of the evidence ranged from low to very low across outcomes. For energy intake, the certainty was rated as low. Although the pooled analysis indicated a significant reduction with no observed heterogeneity, confidence in the estimate was reduced due to concerns related to risk of bias and the relatively small sample size. Certainty was very low for subjective appetite ratings. This was primarily driven by a high risk of bias across most studies, along with imprecision due to limited sample sizes. Similarly, evidence for appetite-related hormones was very low, as it was based on only two small studies, leading to serious concerns about precision.

## 4. Discussion

The present systematic review and meta-analysis examined the effects of post-exercise consumption of milk and milk-based beverages on subjective appetite, appetite-related hormones, and subsequent energy intake in healthy adults. The primary finding was that milk and milk-based beverages were associated with lower post-exercise energy intake compared with CHO beverages. However, subjective appetite ratings were variable, and evidence regarding appetite-related hormones was limited and inconclusive. Notably, the reduction in energy intake was not consistently paralleled by changes in subjective appetite ratings, suggesting that mechanisms beyond conscious appetite perception may contribute to this effect.

### 4.1. Subjective Appetite Responses

Subjective appetite responses following post-exercise consumption of milk and milk-based beverages were variable across studies, with most reporting no significant differences and some indicating modest appetite-suppressing effects compared with CHO-based or non-caloric control beverages. Among the 11 studies included in the systematic review that assessed subjective appetite, only four reported significant differences, indicating lower appetite and higher satiety with milk or milk-based beverages [24,40,42,46]. However, these studies were mostly rated as being at high risk of bias, largely because the absence of blinding is especially relevant for subjective appetite outcomes. Sensory differences between beverages, including taste, texture, thickness, and creaminess, may have influenced appetite ratings through palatability-related or expectancy effects [49,50]. In addition, subjective appetite was frequently treated as a secondary outcome, and sample size calculations were rarely based on these measures, potentially resulting in insufficient statistical power to detect meaningful differences. Therefore, subjective appetite responses should be interpreted cautiously, particularly where blinding was inadequate or studies were not powered for subjective appetite-related outcomes.

Although subjective appetite ratings have been shown to correlate with food intake in some contexts [51,52], this relationship is not consistently observed. In some studies, milk and milk-based beverages reduced subjective appetite ratings without affecting post-exercise total energy intake [24,46], whereas others observed lower energy intake without significant changes in subjective appetite ratings [26,45,48]. This mismatch may occur because subsequent energy intake is shaped by more than conscious appetite sensations. Hormone-mediated satiety signaling and gastric emptying may influence eating behavior beyond conscious appetite perception [53,54]. In parallel, homeostatic and hedonic pathways may differentially regulate food intake depending on physiological need and reward-related responses to food [55]. Protein may also contribute through satiety-related mechanisms, while its thermogenic effects may be more relevant to broader energy balance and weight-management considerations [56]. Therefore, changes in energy intake may occur even when subjective appetite ratings remain unchanged.

Milk has also been shown to influence subjective appetite in non-exercise contexts. A meta-analysis by Onvani et al. (2017) reported that dairy intake significantly reduced hunger, without affecting fullness, desire to eat, or PFC [57]. However, in subgroup analyses, significant effects on fullness, hunger, and PFC emerged only at intakes >500 mL, indicating a dose-dependent relationship [57]. These effects may be partly explained by the protein content of milk, as supported by a meta-analysis showing that acute protein intake significantly reduced hunger, desire to eat, and PFC, while increasing fullness [58]. Although these findings support the potential appetite-modulating properties of milk, their relevance to the post-exercise context remains uncertain.

### 4.2. Appetite-Related Hormonal Responses

Protein has been shown to exert stronger effects on appetite-related hormones than CHO or fat [59,60], suggesting that milk may influence appetite regulation partly through its protein content. However, the evidence in the present systematic review was too limited to support mechanistic conclusions in the post-exercise context. Only two studies assessed hormonal outcomes, with no overlap in the specific hormones measured, resulting in a fragmented and insufficient evidence base [24,48]. Brown et al. (2016) reported higher GLP-1 and insulin levels following a milk-based beverage compared with a non-caloric placebo, but not compared with a CHO beverage [24]. Corney et al. (2023) found no significant differences in acylated ghrelin between beverages, although values were slightly lower following milk consumption [48]. Thus, the hormonal results should be interpreted only as preliminary mechanistic signals, given the very small number of studies and the lack of consistency in the hormones assessed.

Evidence from non-exercise settings suggests that protein intake may influence appetite-related hormones, including GLP-1, ghrelin, and cholecystokinin, with some indication of dose-dependent effects [58,61,62,63]. However, these findings cannot be directly extrapolated to post-exercise milk consumption because of differences in physiological context, intervention composition, and study design. Further well-designed studies with consistent hormonal outcomes are needed before mechanistic conclusions can be drawn regarding the effects of post-exercise milk consumption on appetite-related hormonal responses.

### 4.3. Energy Intake Responses

This systematic review and meta-analysis demonstrated that post-exercise consumption of milk and milk-based beverages was associated with lower energy intake compared with CHO beverages (−72.73 kcal, 95% CI [−141.69; −3.77]). The observed reduction was consistent across analyses and remained robust in sensitivity analyses. Although statistically significant, this acute reduction was modest. While its clinical relevance may be limited for athletes prioritizing post-exercise recovery, repeated small reductions in energy intake may be meaningful in weight-management contexts, as suggested by the small-changes approach to energy balance [64]. However, the acute nature of the included studies prevents direct conclusions regarding long-term effects on habitual energy intake or body weight regulation. Nevertheless, the consistent direction of the acute effect aligns with the meta-analysis by Onvani et al. (2017), which reported reduced energy intake following dairy consumption under non-exercise conditions [57]. Although only 3 of the 10 studies included in our meta-analysis demonstrated a statistically significant reduction in total energy intake compared with CHO beverages [26,45,48], some of the remaining studies also presented noteworthy outcomes. For instance, compared with CHO beverages, milk or milk-based beverages reduced total energy intake by ~188 kcal (~16%) in Brown et al. (2016) [24] and by ~304 kcal (~6%) in Wan et al. (2018) [43]. These differences, while not statistically significant, may still be considered clinically relevant. Indeed, evidence from an expert consensus report suggests that even relatively small reductions in energy intake may contribute to long-term energy balance [64].

Although statistical heterogeneity was not observed in the primary analysis (*I*^2^ = 0%), methodological heterogeneity was present across studies, including differences in exercise modality, beverage composition, protein dose, and beverage volume. Therefore, the pooled estimate should be interpreted as a summary estimate across heterogeneous post-exercise milk-based intervention protocols rather than as evidence of a uniform response across all conditions. Among these sources of methodological variability, differences in beverage administration may have particularly influenced the observed energy intake outcomes. In some studies, post-exercise beverages were provided ad libitum rather than in standardized volumes, introducing variability in fluid and energy intake [25,46]. Although milk and milk-based beverages were associated with reduced ad libitum meal energy intake, inclusion of their energy content in total energy intake shifted the overall balance towards higher energy intake [25,46]. Sensitivity analyses excluding ad libitum beverages protocols showed a slightly larger effect size (−90.87 kcal, 95% CI [−165.77; −18.55], *p* = 0.016), indicating a more pronounced effect under controlled intake conditions. These results suggest that hydration related intake may mask satiety effects in free-living designs and support the use of standardized preload protocols in behavioral nutrition research [65].

The effects of milk on energy intake are largely attributed to its complex nutritional matrix, including proteins/peptides [66], calcium (Ca) [67], lactose/galactose [68], and bioactive peptides [69], as well as its sensory properties such as thickness and creaminess [49,50]. These components may act synergistically, supporting the “dairy matrix” hypothesis, whereby whole-food structures may exert effects beyond isolated nutrients [10]. Consistent with this, Lorenzen et al. (2012) reported ~9% lower energy intake following milk compared with isocaloric beverages containing only whey or casein [70]. Similarly, Gonzalez et al. (2015) reported that neither calcium (3501 ± 253 kJ) nor protein (3699 ± 304 kJ) alone significantly reduced energy intake compared with placebo (4126 ± 395 kJ), whereas their combination resulted in a significant reduction (3419 ± 345 kJ) [71]. In addition, enhanced GLP-1 secretion following combined ingestion of whey protein and calcium-rich milk minerals further supports possible synergistic interactions [72]. Although subgroup analyses in the present meta-analysis did not reveal statistically significant differences between milk (−108.68 kcal, 95% CI [−235.56 to 18.21]) and milk-based beverages (−57.67 kcal, 95% CI [−139.82 to 24.48]), a slightly greater reduction in energy intake was consistently observed with milk, supporting a potential advantage of whole-food dairy structures.

Individual factors likely contribute to variability in energy intake and appetite responses. Sex-related differences in endocrine responses and energy intake behavior have been reported [73,74], although evidence also suggests similar responses between males and females [58,75]. In line with this, subgroup analyses in the present study indicated no clear difference between male (−74.28 kcal, 95% CI [−181.37 to 32.82]) and female (−71.64 kcal, 95% CI [−161.77 to 18.49]) participants. However, the limited evidence base and lack of mixed-sex studies restrict interpretation. Beyond sex, cognitive and behavioral factors may also be relevant. Virgilio et al. (2019) reported that milk-based beverages decreased energy intake by 10%, but only women with higher levels of cognitive dietary restraint, with no significant differences detected in the overall sample [47].

Dietary interventions may reduce appetite and energy intake in the short term, but such effects are not always maintained over longer durations [58,76]. All studies included in this meta-analysis used acute designs, limiting extrapolation to long-term adaptations. Although longitudinal studies (4.5 to 12 weeks) have examined the effects of post-exercise milk consumption on body composition, these studies were not eligible for inclusion in the present systematic review [77,78,79,80]. This is because dietary assessments were primarily used to standardize intake between visits and to monitor adherence to the study protocol, rather than to evaluate appetite regulation or post-exercise energy intake. Moreover, the structure of dietary records often made it unclear whether intake reflected post-exercise periods, particularly because exercise was performed only on selected days of the week and records did not consistently distinguish exercise from non-exercise days. Participants were also sometimes instructed to maintain or replicate habitual dietary patterns, which may have introduced additional variability [79,80]. Consequently, it remains unclear whether the observed acute reduction in energy intake would persist over time, particularly under free-living conditions, across repeated exercise sessions, or within habitual dietary patterns.

### 4.4. Limitations and Implications for Future Research

Several limitations should be considered when interpreting the findings of this systematic review and meta-analysis. Study protocols varied in exercise modality, exercise volume and duration, timing of outcome assessments, participant characteristics, beverage composition, comparator type, timing, and dosage. The relatively small number of studies and participants suggests that the pooled analysis should be viewed as preliminary, particularly because the available evidence is estimated from small, acute trials with heterogeneous protocols. Furthermore, meta-analysis was not feasible for subjective appetite ratings or appetite-related hormones due to substantial heterogeneity in outcome measurement and reporting. Even for energy intake, methodological differences across studies and the low certainty of evidence limit confidence in the observed effects.

Limitations were also identified at the level of the included randomized controlled trials. Blinding was often not feasible due to differences in beverage sensory characteristics, which may have introduced bias, particularly for subjective outcomes. In addition, studies focused primarily on changes in energy intake without evaluating whether post-exercise nutritional recommendations for recovery were met. Appetite-related hormones were assessed in only a small number of studies, and other relevant physiological mechanisms, such as gastric emptying rate, were not evaluated. In some studies, intervention and comparator beverages were not matched for volume or energy content, which may have influenced subsequent energy intake. Beverage temperature, which has been reported to influence energy intake [81,82], was not reported in several studies. Participant-related factors also remain important, particularly dietary restraint, which may influence appetite and energy intake responses but was rarely assessed or used as an exclusion criterion.

Future research should focus on well-controlled randomized trials with standardized protocols, matching beverage energy content, volume, and temperature. Studies should also include more consistent assessment of appetite-related hormones and relevant mechanisms, using both acute and longer-term designs. Better characterization of participant traits, particularly dietary restraint, would help clarify inter-individual variability. Finally, although no significant differences were observed between milk and milk-based beverages, the slightly greater reduction in energy intake following milk suggests that the whole dairy matrix may warrant further investigation in relation to appetite and energy regulation.

## 5. Conclusions

Post-exercise consumption of milk and milk-based beverages was associated with lower subsequent energy intake compared with active controls (i.e., CHO beverages); however, the certainty of the evidence is low, primarily due to risk of bias and imprecision. This finding suggests that milk may extend its role beyond recovery by contributing to the maintenance of an exercise-induced negative energy balance. This effect may be particularly relevant for athletes seeking to modulate body weight or body fat. The mechanisms underlying the observed effects on energy intake remain unclear and likely reflect the combined influence of gastrointestinal hormones, gastric emptying, protein-related effects, and sensory characteristics of the beverages.

## Figures and Tables

**Table 1:** Eligibility criteria based on the PICOS framework.

	Inclusion	Exclusion
Population	Healthy adults (≥18 years), including trained or untrained individuals, regardless of sex or body mass status (normal weight or overweight)	Participants with diagnosed chronic diseases or clinical conditions
Intervention	Post-exercise consumption of milk, milk-based beverages, or milk protein beverages (e.g., whey or casein beverages)	Studies investigating consumption before or during exercise, or interventions not involving milk or milk-based beverages
Comparator	Carbohydrate drinks, sports drinks, placebo beverages, or other active control beverages	Studies without a comparator or studies comparing only different milk-based beverages
Outcome	Subjective appetite ratings, appetite-related hormones, or post-exercise energy intake	Studies not reporting appetite-related outcomes or energy intake
Study design	Randomized study designs, including randomized controlled trials, randomized crossover trials, and randomized parallel-group trials	Non-randomized studies, observational studies, animal studies, and in vitro studies

**Table 2:** Characteristics of included studies examining the effects of post-exercise milk or milk-based beverages on appetite regulation.

Author (Year)	Participants	Exercise Type	Post-Exercise Beverages	Outcomes		
				Energy Intake	Subjective Appetite Ratings	Appetite-Related Hormones
Desbrow (2014) [40]	Recreationally active males (*n* = 15) 24.9 ± 5.5 y	Cycling (~60-min at 70–80% HRmax to ~1.8% body mass loss)	Milk (~2.2 L, ~1504 kcal, ~79.2 g protein) MBB (~2.2 L, ~2193 kcal, ~143 g protein) SM (~2.2 L, ~1435 kcal, ~70.4 g protein) CB (~2.2 L, ~678 kcal, 0 g protein)	NA	Hunger: CB > others, Milk > MBB and SM Fullness: MBB > others, Milk and SM > CB	NA
Clayton (2014) [41]	Recreationally active males (*n* = 12) 24 ± 2 y V̇O_2_peak: 52 ± 8 mL/kg/min	Cycling (30-min at ~60% V̇O_2_peak + 5 × 3-min intervals at ~85% V̇O_2_peak)	WB (500 mL, 126 kcal, 30.3 g protein) CB (500 mL, 126 kcal, 0.6 g protein) Placebo (500 mL, 3.6 kcal, 0.3 g protein)	Ad libitum (meal): WB < Placebo Total: ↔	Hunger, fullness, PFC, and desire to eat: ↔	NA
Rumbold (2015) [26]	Recreationally active females (*n* = 9) 19.7 ± 1.3 y V̇O_2_peak: 45.7 ± 13.4 mL/kg/min	Cycling (30-min at ~65% V̇O_2_peak)	Milk (600 mL, 210 kcal, 20.4 g protein) CB (600 mL, 210 kcal, 2.4 g protein)	Ad libitum (meal): Milk < CB	Hunger, fullness, and PFC: ↔	NA
Seery (2016) [42]	Active males (*n* = 7) 26.2 ± 6.1 y	Cycling (50-min cycling in heat + 10-min intervals to ~1.8% body mass loss, total duration: ~100 min)	Milk (~2732 mL, ~947 kcal, ~90 g protein) CB (~2796 mL, ~468 kcal, 0 g protein) Placebo (~2764 mL, 0 kcal, 0 g protein)	NA	Hunger: Milk < CB and Placebo	NA
Brown (2016) [24]	Recreationally active females (*n* = 13) 23 ± 4 y V̇O_2_peak: 43.5 ± 11.6 mL/kg/min	Cycling (30-min at ~65% V̇O_2_peak)	MBB (524 mL, 325 kcal, 22.2 g protein) CB (524 mL, 325 kcal, <0.5 g protein) Placebo (524 mL, 0 kcal, 0 g protein)	Ad libitum (meal): MBB < Placebo, MBB ↔ CB Free-living: ↔ Total: ↔	Hunger: MBB < Placebo, MBB ↔ CB Fullness: MBB > Placebo, MBB ↔ CB PFC: MBB < Placebo, MBB ↔ CB	GLP-1: MBB > Placebo, MBB ↔ CB Insulin: MBB > Placebo, MBB ↔ CB, Leptin: ↔
Campagnolo (2017) [25]	Trained males (*n* = 10) 25.3 ± 4.9 y V̇O_2_max: 63.0 ± 7.2 mL/kg/min	Cycling (~60-min at 65% PPO to ~1.8% body mass loss)	MBB (~1776 mL, ~1770 kcal, ~115 g protein) CB (~2651 mL, ~812 kcal, 0 g protein) Placebo 1 (~2121 mL) and Placebo 2 (~2076 mL) (both 0 kcal and 0 g protein)	Ad libitum (beverages): MBB > others Ad libitum (meal): MBB < others Total: MBB > Placebo 1 and 2, MBB ↔ CB	Hunger and fullness: ↔	NA
Wan (2018) [43]	Trained males (*n* = 12) 21 ± 1 y V̇O_2_max: 63.0 ± 4.1 mL/kg/min	Cycling (2-min bouts at 90% Pmax + 2-min recovery at 50% Pmax, with 10% decrements to failure, total duration: ~100 min)	Milk (~1020 mL, ~765 kcal, ~38 g protein) CB (~1020 mL, ~213 kcal, 0 g protein)	Free-living: ↔ Total: ↔	NA	NA
Maltais (2018) [44]	Physically active sarcopenic males (*n* = 9) 64 ± 3 y	Resistance (60-min, 3 series of 4 different exercises, 1 set × 8 repetitions per exercise at ~75% 1RM)	Milk (375 mL, 270 kcal, 13 g protein) RM (395 mL, 280 kcal, 0.6 g protein) Placebo (0 kcal, 0 g protein)	Ad libitum (meal): ↔ Free-living: ↔ Total: ↔	Hunger, fullness, PFC, and desire to eat: ↔	NA
Monteyne (2018) [45]	Active males (*n* = 15) 21 ± 1 y	Resistance (~50-min, unilateral knee extension/flexion of both legs, 4 sets × 10 repetitions at 70% 1RM)	WB (500 mL, ~110 kcal, ~23.9 g protein) CB (500 mL, ~110 kcal, ~0.4 g protein)	Ad libitum (meal): WB < CB	Hunger, fullness, PFC, and desire to eat: ↔	NA
McCartney (2019) [46]	Trained females (*n* = 8) 33.2 ± 7.4 y V̇O_2_max: 46.3 ± 7.5 mL/kg/min	Cycling (~60-min at ~60% PPO to ~1.8% body mass loss)	Milk (~1538 mL, ~1023 kcal, ~52 g protein) HP-Milk (~1639 mL, ~1348 kcal, ~110 g protein) CB (~1801 mL, ~448 kcal, 0 g protein) Placebo (~1790 mL, 0 kcal, 0 g protein)	Ad libitum (beverages): Milk ↔ HP-Milk > CB > Placebo Ad libitum (meal): Milk < Placebo, HP milk < Placebo ↔ CB, Milk ↔ HP-Milk Free-living: ↔ Total: ↔	Hunger: Milk and HP-Milk < CB and Placebo (only 1st hour of recovery) Fullness: Milk and HP-Milk > Placebo (only 1st hour of recovery)	NA
Virgilio (2019) [47]	Moderately active females (*n* = 20) 24 ± 7 y 600–1500 MET-min/week	Not specified aerobic exercise	MBB (250 mL, ~117.5 kcal, ~23.25 g protein) CB (250 mL, ~117.5 kcal, ~0.75 g protein)	Free-living: ↔	Hunger, fullness, PFC, and desire to eat: ↔	NA
Corney (2023) [48]	Active males (*n* = 10) 21.3 ± 1.2 y V̇O_2_peak: 58 ± 5 mL/kg/min	Cycling (30-min at ~60% V̇O_2_peak + 5 × 4-min intervals at ~85% V̇O_2_peak)	Milk (615 mL, 239 kcal, 21.6 g protein) CB (615 mL, 239 kcal, 0 g protein) Placebo (615 mL, 14 kcal, 0 g protein)	Ad libitum (meal): Milk < CB, Milk ↔ Placebo Total: Milk < CB, Milk ↔ Placebo	Hunger and fullness: ↔	Acylated ghrelin: ↔

↔: not significant (*p* > 0.05), >: significantly higher (*p* < 0.05), <: significantly lower (*p* < 0.05), 1RM: one-repetition maximum, CB: carbohydrate beverage, GLP-1: glucagon-like peptide-1, HP-Milk: high protein milk, HRmax: maximum heart rate, MBB: milk-based beverage, MET: metabolic equivalent of task, NA: not available, PFC: prospective food consumption, Pmax: maximal power output, PPO: peak power output, RM: rice milk, SM: soy milk, V̇O_2_max: maximal oxygen uptake, V̇O_2_peak: peak oxygen uptake, WB: whey beverage.

**Table 3:** Summary of findings.

Outcomes	Estimated Absolute Effects		№ of Participants (Studies)	Certainty of the Evidence (GRADE) *	Comments
	Mean Difference	Relative Effect (95% CI)			
Energy intake	−72.73 kcal	−141.69 to −3.77	118 (10 RCTs)	⨁⨁◯◯ Low ^a,b^	Moderate but significant reduction
Subjective appetite ratings	Not estimable		125 (11 RCTs)	⨁◯◯◯ Very low ^b,c^	No consistent effect; effects (when present) generally favored appetite suppression; high heterogeneity and incomplete reporting
Appetite-Related Hormones	Not estimable		23 (2 RCTs)	⨁◯◯◯ Very low ^a,d^	Very limited evidence; no synthesis possible

* Certainty of evidence according to Grading of Recommendations, Assessment, Development and Evaluations (GRADE): High: we are very confident that the true effect lies close to that of the estimate of the effect. Moderate: we are moderately confident in the effect estimate: the true effect is likely to be close to the estimate of the effect, but there is a possibility that it is substantially different. Low: our confidence in the effect estimate is limited: the true effect may be substantially different from the estimate of the effect. Very low: we have very little confidence in the effect estimate: the true effect is likely to be substantially different from the estimate of effect. Reasons for downgrading certainty of the evidence: ^a^ Downgraded by one level because several studies were judged as having some concerns according to the RoB 2 tool. ^b^ Downgraded by one level due to small sample sizes and limited total numbers of participants. ^c^ Downgraded by two levels for risk of bias, as most studies were rated at high risk according to the RoB 2 tool. ^d^ Downgraded by two levels for very limited evidence base (only 2 small studies available).

## Data Availability

Data will be made available on request.

## Supplementary Materials

The following supporting information can be downloaded at: https://www.mdpi.com/article/10.3390/nu18111656/s1, Prisma Checklist; Supplementary Materials S1 (Search Plan); Figure S1: Forest plot of subgroup analysis by sex comparing energy intake between milk/milk-based beverage and carbohydrate-based control groups; Figure S2: Forest plot of subgroup analysis by intervention type (milk vs. milk-based beverage) comparing energy intake; Figure S3: Funnel plot for the assessment of publication bias across the included studies.

## Abbreviations

The following abbreviations are used in this manuscript:

1RM	One-repetition maximum
Ca	Calcium
CB	Carbohydrate beverage
CHO	Carbohydrate
CI	Confidence intervals
*df*	Degrees of freedom
GLP-1	Glucagon-like peptide-1
HP-Milk	High protein milk
HRmax	Maximum heart rate
kcal	Kilocalories
MBB	Milk-based beverage
MD	Mean differences
MET	Metabolic equivalent of task
NA	Not available
PFC	Prospective food consumption
Pmax	Maximal power output
PPO	Peak power output
RM	Rice milk
SD	Standard deviations
SEM	Standard errors of the mean
SM	Soy milk
SMD	Standardized mean differences
V̇O_2_max	Maximal oxygen uptake
V̇O_2_peak	Peak oxygen uptake
WB	Whey beverage
