# Supplementary material for: Beyond Recovery: Effects of Post-Exercise Milk and Milk-Based Beverages on Appetite Regulation and Energy Intake—A Systematic Review and Meta-Analysis
Source: Nutrients. 2026 May 22;18(11):1656. doi: 10.3390/nu18111656 (PMC13257937; doi:10.3390/nu18111656)
Supplement: Supplementary file 1 [file nutrients-18-01656-s001.zip › Search Plan.pdf]

milk	"Milk"[Mesh]
dairy	"Dairy Products"[Mesh]
Milk protein	"Milk protein"[TW]
Whey	"Whey"[Mesh]
protein	"Proteins"[Mesh] OR "Protein*”[TW]

AND

Exercise	"Exercise"[Mesh]
Post exercise	“Post exercise"[TW] OR “Post-exercise"[TW]
Exertion	“Exertion"[TW]
fitness	“Fitness"[TW]
Activity	“Activ*"[TW]
Sport	"Sports"[Mesh] OR “Sport*”[TW]
Training	“Train*”[TW]
Recovery	"Post-Exercise Recovery"[Mesh]
workout	“Workout"[TW]

AND

Appetite	"Appetite"[Mesh] OR "Appetite Regulation"[Mesh]
Satiety	“Satiety"[TW]
Hunger	"Hunger"[Mesh]
Fullness	“Fullness"[TW]
Energy intake	"Energy Intake"[Mesh]
Energy balance	“Energy Balance"[TW]

Pubmed: 04.04.2025 / 1200

(("Milk"[Mesh] OR "Dairy Products"[Mesh] OR "Milk protein"[TW] OR "Whey"[Mesh] OR "Proteins"[Mesh] OR "Protein*"[TW]) AND ("Exercise"[Mesh] OR "Post exercise"[TW] OR "Post-exercise"[TW] OR "Exertion"[TW] OR "Fitness"[TW] OR "Activ*"[TW] OR "Sports"[Mesh] OR "Sport*"[TW] OR "Train*"[TW] OR "Post-Exercise Recovery"[Mesh] OR "Workout"[TW])) AND ("Appetite"[Mesh] OR "Appetite Regulation"[Mesh] OR "Satiety"[TW] OR "Hunger"[Mesh] OR "Fullness"[TW] OR "Energy Intake"[Mesh] OR "Energy Balance"[TW]) AND ((adaptiveclinicaltrial[Filter] OR clinicalstudy[Filter] OR clinicaltrial[Filter] OR controlledclinicaltrial[Filter] OR randomizedcontrolledtrial[Filter] OR randomizedcontrolledtrialveterinary[Filter]) AND (humans[Filter])) AND ((adaptiveclinicaltrial[Filter] OR clinicalstudy[Filter] OR clinicaltrial[Filter] OR controlledclinicaltrial[Filter] OR randomizedcontrolledtrial[Filter]) AND (humans[Filter]))

Web of Science: 04.04.2025 / 2316

(TS=((milk OR “dairy products" OR "milk protein" OR whey OR protein*) AND (exercise OR “post exercise" OR “post-exercise" OR exertion OR fitness OR activ* OR sport* OR train* OR "post-exercise recovery" OR workout) AND (appetite OR “appetite regulation" OR satiety OR hunger OR fullness OR “energy intake" OR “energy balance"))) AND TS=(intervention OR "intervention* stud*" OR "controlled trial*" OR random* OR placebo OR clinical OR "clinical trial*" OR trial* OR "randomi?ed controlled trial*" OR "randomi?ed clinical trial*" OR RCT OR blinded OR “double-blind*" OR "double blind*" OR "Cross over " OR "Cross-Over" OR "parallel study" OR "parallel trial") and Article (Document Types)

Cochrane Library (Central): 04.04.2025 / 1615
((Milk[Mesh] OR "Dairy Products"[Mesh] OR "Milk protein" OR Whey[Mesh] OR Proteins[Mesh] OR Protein*) AND (Exercise[Mesh] OR "Post exercise" OR "Post-exercise" OR Exertion OR Fitness OR Activ* OR Sports[Mesh] OR Sport* OR Train* OR "Post-Exercise Recovery"[Mesh] OR Workout) AND (Appetite[Mesh] OR "Appetite Regulation"[Mesh] OR Satiety OR Hunger[Mesh] OR Fullness OR "Energy Intake"[Mesh] OR "Energy Balance")) in Trials

Scopus: 05.04.2025 / 1078
((TITLE-ABS-KEY (milk) OR TITLE-ABS-KEY ("dairy products") OR TITLE-ABS-KEY ("milk protein") OR TITLE-ABS-KEY (whey) OR TITLE-ABS-KEY (ptotein*))) AND ((TITLE-ABS-KEY (exercise) OR TITLE-ABS-KEY ("post exercise") OR TITLE-ABS-KEY ("post-exercise") OR TITLE-ABS-KEY (exertion) OR TITLE-ABS-KEY (fitness) OR TITLE-ABS-KEY (activ*) OR TITLE-ABS-KEY (sport*) OR TITLE-ABS-KEY (train*) OR TITLE-ABS-KEY ("post-exercise recovery") OR TITLE-ABS-KEY (workout))) AND ((TITLE-ABS-KEY (appetite) OR TITLE-ABS-KEY ("appetite regulation") OR TITLE-ABS-KEY (satiety) OR TITLE-ABS-KEY (hunger) OR TITLE-ABS-KEY (fullness) OR TITLE-ABS-KEY ("energy intake") OR TITLE-ABS-KEY ("energy balance"))) AND ((TITLE-ABS-KEY (intervention) OR TITLE-ABS-KEY ("intervention* stud*") OR TITLE-ABS-KEY ("controlled trial*") OR TITLE-ABS-KEY (random*) OR TITLE-ABS-KEY (placebo) OR TITLE-ABS-KEY (clinical) OR TITLE-ABS-KEY ("clinical trial*") OR TITLE-ABS-KEY (trial*) OR TITLE-ABS-KEY ("randomi?ed controlled trial*") OR TITLE-ABS-KEY ("randomi?ed clinical trial*") OR TITLE-ABS-KEY (rct) OR TITLE-ABS-KEY (blinded) OR TITLE-ABS-KEY ("double-blind*") OR TITLE-ABS-KEY ("double blind*") OR TITLE-ABS-KEY ("Cross over") OR TITLE-ABS-KEY ("Cross-Over") OR TITLE-ABS-KEY ("parallel study") OR TITLE-ABS-KEY ("parallel trial")))

Ovid MEDLINE ALL: 06.04.2025 / 967
(((Milk or "Dairy Products" or "Milk protein" or Whey or Protein*) and (Exercise or "Post exercise" or "Post-exercise" or Exertion or Fitness or Activ* or Sport* or Train* or "Post-Exercise Recovery" or Workout) and (Appetite or "Appetite Regulation" or Satiety or Hunger or Fullness or "Energy Intake" or "Energy Balance") and (intervention or "intervention* stud*" or "controlled trial*" or random* or placebo or clinical or "clinical trial*" or trial* or "randomi?ed controlled trial*" or "randomi?ed clinical trial*" or RCT or blinded or "double-blind*" or "double blind*" or "Cross over " or "Cross-Over" or "parallel study" or "parallel trial")).ab.)limit:humans

EBSCO Open Dissertation: 06.04.2025 / 106

(Milk OR “Dairy Products" OR "Milk protein" OR Whey OR Protein*) AND (Exercise OR “Post exercise" OR “Post-exercise" OR Exertion OR Fitness OR Activ* OR Sport* OR Train* OR "Post-Exercise Recovery" OR Workout) AND (Appetite OR “Appetite Regulation" OR

Satiety OR Hunger OR Fullness OR “Energy Intake" OR “Energy Balance") AND (intervention OR "intervention* stud*" OR "controlled trial*" OR random* OR placebo OR clinical OR "clinical trial*" OR trial* OR "randomi?ed controlled trial*" OR "randomi?ed clinical trial*" OR RCT OR blinded OR “double-blind*" OR "double blind*" OR "Cross over" OR "Cross-Over" OR "parallel study" OR "parallel trial")

Open Access Theses and Dissertations (OATD): 06.04.2025 / 249

(((((milk) OR ("dairy product") OR ("milk protein") OR (whey) OR (protein*)) AND ((exercise) OR ("Post exercise") OR ("post-exercise") OR (Exertion) OR (Fitness) OR (Activ*) OR (Sport*) OR (Train*) OR ("Post-Exercise Recovery") OR (Workout))) AND ((appetite) OR ("Appetite Regulation") OR (Satiety) OR (Hunger) OR (Fullness) OR ("Energy Intake") OR ("Energy Balance"))) AND ((intervention) OR ("intervention* stud*") OR ("controlled trial*") OR (random*) OR (placebo) OR (clinical) OR ("clinical trial*") OR (trial*) OR ("randomised controlled trial*") OR ("randomized controlled trial*") OR ("randomised clinical trial*") OR ("randomized clinical trial*") OR (RCT) OR (blinded) OR ("double-blind*") OR ("double blind*") OR ("Cross over") OR ("Cross-Over") OR ("parallel study") OR ("parallel trial")))
