# Supplementary material for: Beyond Recovery: Effects of Post-Exercise Milk and Milk-Based Beverages on Appetite Regulation and Energy Intake—A Systematic Review and Meta-Analysis
Source: Nutrients. 2026 May 22;18(11):1656. doi: 10.3390/nu18111656 (PMC13257937; doi:10.3390/nu18111656)
Supplement: Supplementary file 1 [file nutrients-18-01656-s001.zip › Supplementary Figures.pdf]

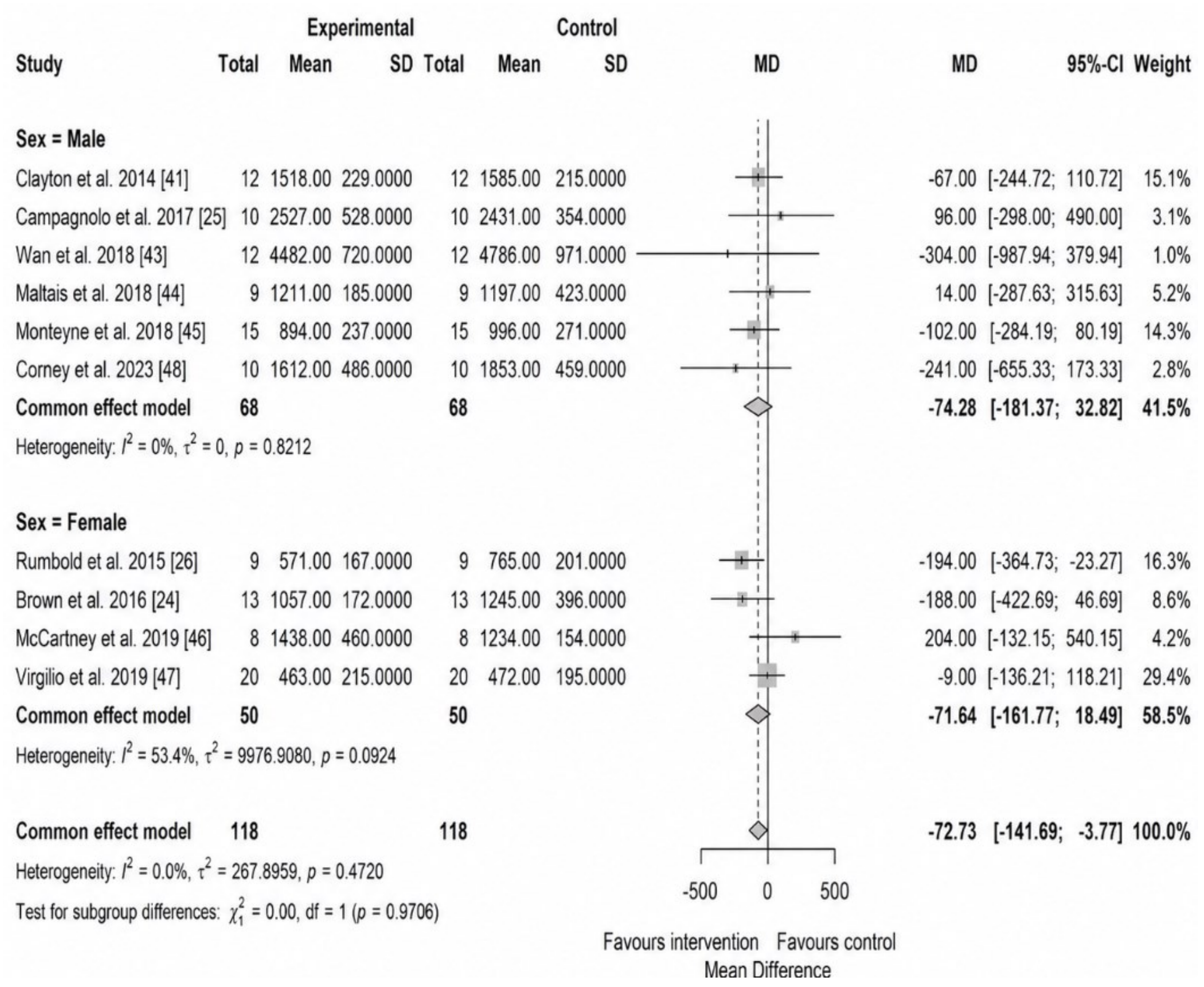

Figure S1. Forest plot of subgroup analysis by sex comparing energy intake between milk/milk-based beverage and carbohydrate-based control groups.

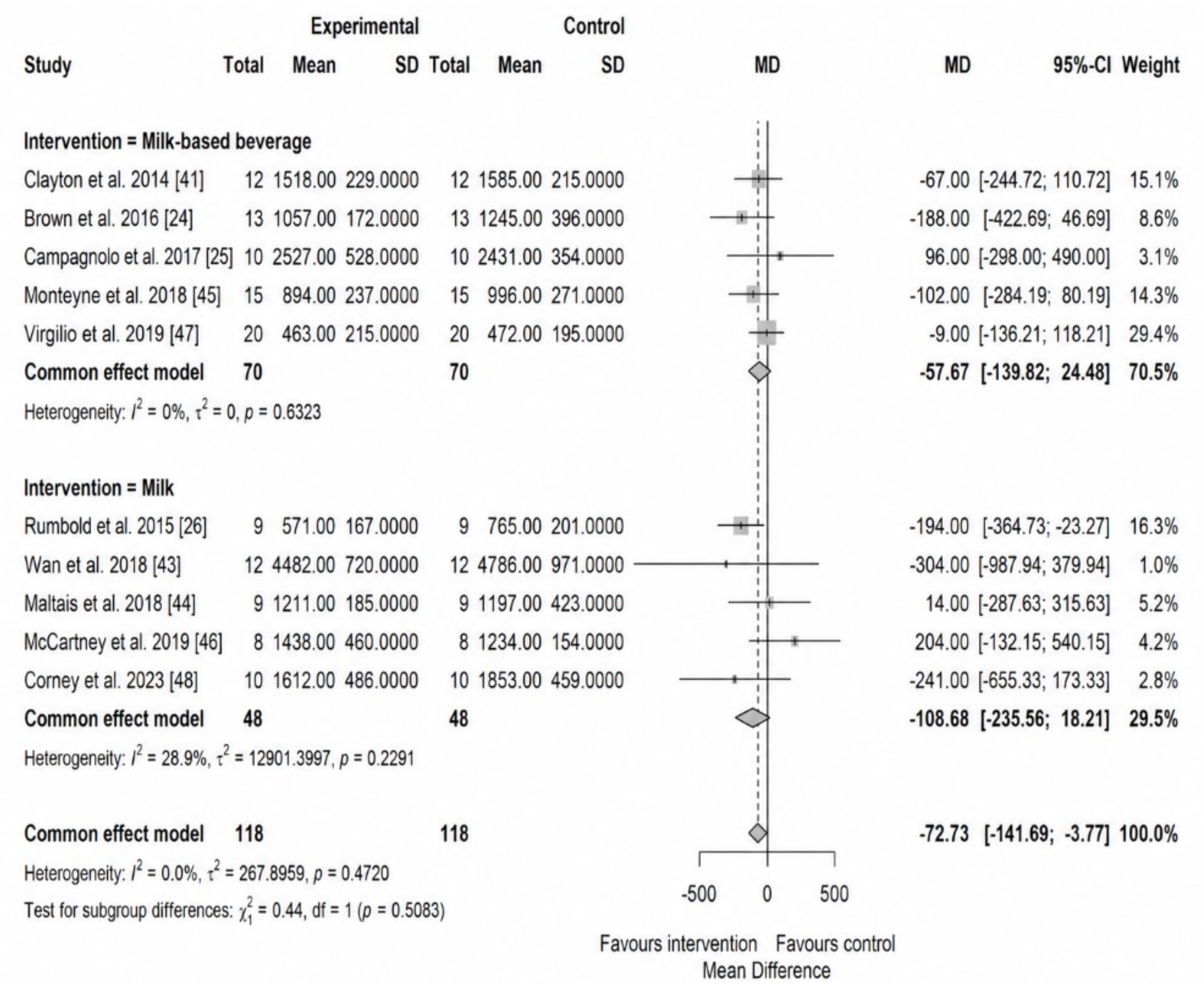

Figure S2. Forest plot of subgroup analysis by intervention type (milk vs. milk-based beverage) comparing energy intake.

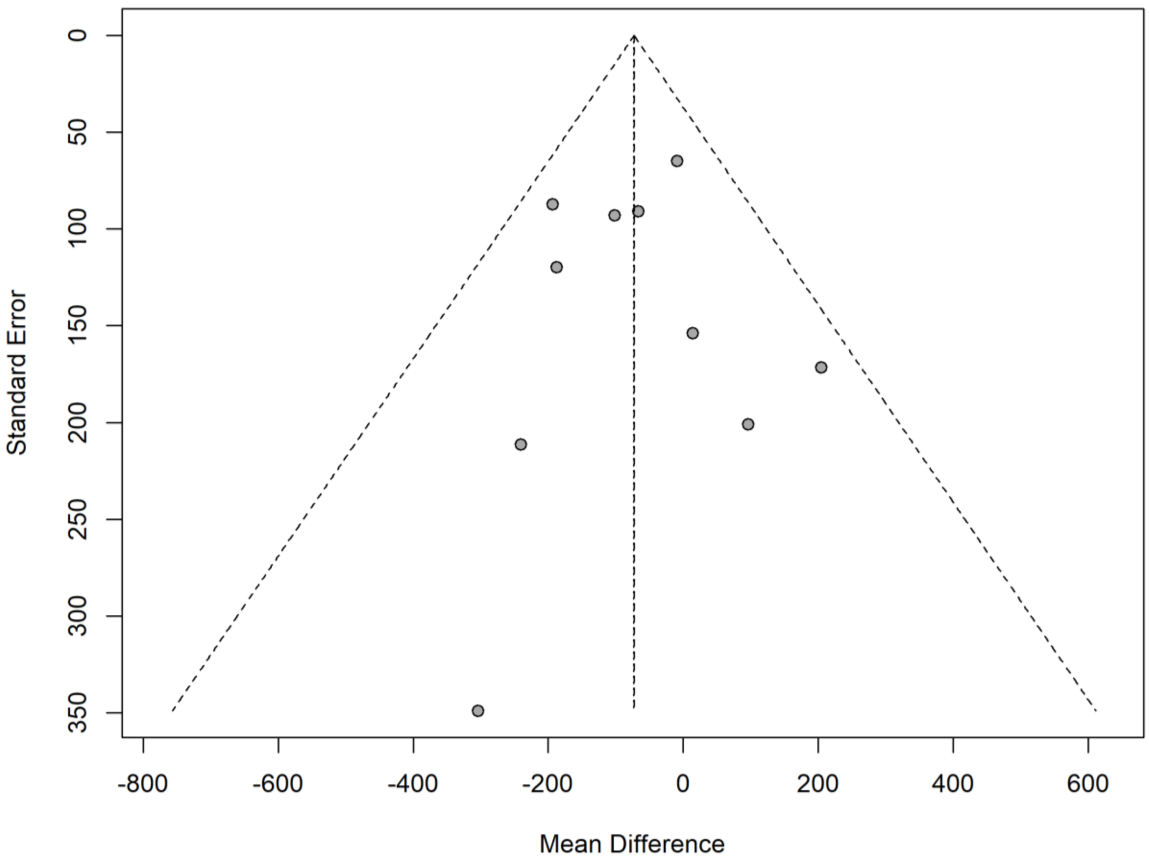

Figure S3. Funnel plot for the assessment of publication bias across the included studies.
